# Clustering of *Mycobacterium tuberculosis* Cases in Acapulco: Spoligotyping and Risk Factors

**DOI:** 10.1155/2011/408375

**Published:** 2010-12-08

**Authors:** Elizabeth Nava-Aguilera, Yolanda López-Vidal, Eva Harris, Arcadio Morales-Pérez, Steven Mitchell, Miguel Flores-Moreno, Ascencio Villegas-Arrizón, José Legorreta-Soberanis, Robert Ledogar, Neil Andersson

**Affiliations:** ^1^Centro de Investigación de Enfermedades Tropicales (CIET), Universidad Autónoma de Guerrero, Calle Pino S/N, Colonia El Roble, 39640 Acapulco, Guerrero, Mexico; ^2^Departamento de Microbiología y Parasitología, Facultad de Medicina, Universidad Nacional Autónoma de México (UNAM), Circuito Escolar S/N, Coyoacán, 04510 Mexico City, Mexico; ^3^Division of Infectious Diseases, School of Public Health, University of California, Berkeley, 1 Barker Hall, Berkeley, CA 94720-7354, USA; ^4^CIET Canada, Institute of Population Health, University of Ottawa, 319-1 Stewart Street, Ottawa, Canada K1N 6N5

## Abstract

Recurrence and reinfection of tuberculosis have quite different implications for prevention. We identified 267 spoligotypes of *Mycobacterium tuberculosis* from consecutive tuberculosis patients in Acapulco, Mexico, to assess the level of clustering and risk factors for clustered strains. Point cluster analysis examined spatial clustering. Risk analysis relied on the Mantel Haenszel procedure to examine bivariate associations, then to develop risk profiles of combinations of risk factors. Supplementary analysis of the spoligotyping data used SpolTools. Spoligotyping identified 85 types, 50 of them previously unreported. The five most common spoligotypes accounted for 55% of tuberculosis cases. One cluster of 70 patients (26% of the series) produced a single spoligotype from the Manila Family (Clade EAI2). The high proportion (78%) of patients infected with cluster strains is compatible with recent transmission of TB in Acapulco. Geomatic analysis showed no spatial clustering; clustering was associated with a risk profile of uneducated cases who lived in single-room dwellings. The Manila emerging strain accounted for one in every four cases, confirming that one strain can predominate in a hyperendemic area.

## 1. Introduction

Tuberculosis (TB) remains a global health problem, mainly related to poverty and concomitant diseases [1]. The reported annual incidence of pulmonary TB in Mexico is 14.27 cases per 100,000 people; in Guerrero state, the rate is 34.56 per 100,000 [2]. Acapulco is of particular concern as a centre for internal migration in Guerrero state, in addition to its role in international tourism. 

TB recurrence and reinfection have quite different implications for prevention. A key issue among migrants to a city like Acapulco is whether TB infection contracted earlier in life is reactivated, to be detected in the city as clinical cases, or whether these are new infections contracted in the city. One research device to help differentiate between recurrence and reinfection is to establish whether contemporary cases are clustered; unless they all came from the same distant place of original infection, clustering suggests a shared source of infection in the locality of the study.

Molecular typing is a well-recognized tool for identifying clustering. The sequence insertion of IS6110 is often the basis for molecular fingerprinting, as the number of copies of this insertion and its localization in the chromosome varies among different strains [6]. Another sequence targeted for molecular typing is the 36-base pair Direct Repeat (DR) locus. Spoligotyping is a PCR-based typing method that generates distinct patterns based on the hybridization of 43 different oligonucleotides to amplified spacer sequences that lie between DR loci in the *M. tuberculosis* chromosome [7]. This estimates the frequency of recent infection with *M. tuberculosis* estimated by identification of clusters of a shared spoligotype. 

This paper studies spoligotypes isolates of *M. tuberculosis* from a series of patients in Acapulco, to determine the level of clustering and to identify risk factors for clustering.

## 2. Material and Methods

### 2.1. Study Location and Population

A total of 330 patients with pulmonary TB presented a positive diagnosis between February 2001 and September 2002 in the municipality of Acapulco. Receipt of funding set the start of the series which closed with 330 consecutive participants recruited through a review of recent medical records. The series excluded only 30 who had left the city and who could not be localised. Using addresses from clinical records, researchers approached each participant and explained the study, obtaining written consent prior to administering a pretested questionnaire. The study provided an opportunity to follow patients with a preliminary sputum positive result; the Ministry of Health offered directly observed therapy, short course strategy (DOTS) [8] for all cases.

### 2.2. Bacterial Strains

The 300 participants contacted yielded 273 isolates of *M. tuberculosis *from sputum samples collected by the Acapulco General Hospital, the Donato G. Alarcón Hospital, the State Public Health Laboratory and the Clínica Avanzada de Atención Primaria a la Salud. Processing began with the Petroff method for preparation and decontamination of sputum samples, followed by inoculation of 0.5 mL of each sample onto Löwenstein-Jensen media, incubated at 37°C for eight weeks.

### 2.3. Genotyping

Extraction of DNA required resuspension of *M. tuberculosis* colonies in 1 mL of sterile distilled water, with samples lysed at 90°C for 10 minutes and frozen at −70°C for 15 minutes. Samples were then thawed and incubated at 90°C for 10 minutes. Centrifugation for three minutes concentrated bacterial contents, and the supernatant was transferred to a new tube. Spoligotyping relied on a commercially available kit (Isogen Bioscience BV Maarssen, The Netherlands) for amplification of DNA from the DR locus, the region with the highest level of polymorphism in the *M. tuberculosis* chromosome [7]. PCR amplification used 5 uL of extracted DNA from each sample combined with 1.5 uL MgCl_2_, 2.5 mM of each dNTP, 5 *μ*l 10X buffer, 0.5 U Taq polymerase, and 20 pmol of each primer (DRa: biotin-5′-CCG AGA GGG GAC GGA AAC-3′ and DRb: 5′-GGT TTT GGG TCT GAC GAC-3′) in a final volume of 50 uL. The amplification protocol required 5 minutes at 94°C followed by 30 cycles of 1 minute at 94°C, 1 minute at 55°C, and 30 seconds at 72°C, with a final extension of 10 minutes at 72°C. The assay included two positive controls (chromosomal DNA from *M. tuberculosis* H37Rv and from *M. bovis* BCG P3) and a negative control (sterile H_2_O).

We hybridized the amplified DNA with 43 oligonucleotides covalently linked to a nylon membrane (Isogen Bioscience BV, Maarssen, The Netherlands) at 60°C for 1 hour in a blotter with 45 lines (Miniblotter 45; Immunetics, Cambridge, Mass). Afterwards, hybridized DNA fragments were incubated with streptavidin, conjugated to peroxidase (Boehringer Mannheim), and then analyzed by chemi-luminescence by incubating for 1 minute in 20 mL of ECL detection reagent (Amersham, Buckinghamshire, England) and exposing to X-ray film for 20 minutes.

### 2.4. Data Capture and Analysis

Data capture from the questionnaires relied on Epi-Info (CDC, version 6.04). We compared spoligotyping results with the SpolDB4 spoligotyping database from the Institute Pasteur de Guadeloupe which, at the time of the analysis, contained 1,939 types from 39,295 strains contributed by 122 countries [3, 9]. The analysis defined a cluster as two or more isolates with identical genetic patterns. We used the preexisting code for those already identified matching patterns. When the spoligotype was not found in SpolDB4, we labeled it as “Mx” with a number (for example, Mx1).

We estimated the Recent Transmission Index (RTI) using the formula of RTI*n* − 1 [10] and RTI*n*, which takes into account the number of patterns with unique genotype (singletons) described by Luciani et al. [11]. Supplementary analysis of the spoligotyping data used SpolTools [12]: DESTUS (Detecting Emerging Strains of Tuberculosis Using Spoligotypes) focused on the detection of rapidly propagating strains; spoligoforests allowed visualization of probable relations between the spoligotypes based on a plausible history of mutation events. The clusters of strains sharing the same spoligotype in the diagram are nodes, labeled with shared type (ST) numbers in SpolDB4 [3].

### 2.5. Risk and Point Cluster Analysis

Potential risk factors for clustering included age (less than or older than 30 years), sex, marital status, education, employment, ethnicity, area of residence (urban or rural), duration of residence, number of people in the dwelling, use of alcohol, concomitant illness, or spouse with TB. When simple bivariate risk analysis revealed no statistically significant contrasts, we derived risk profiles of factor combinations associated with clustering. We examined these risk profiles in a multivariate analysis, beginning with a saturated model and eliminating the weakest association stepwise until only significant associations remained in the final model. We report this as an adjusted odds ratio and 95% confidence interval. 

For the spatial analysis, we geoindexed cases on a map of Acapulco at a topographic chart scale of 1 : 50,000 in DXF vector data format (Instituto Nacional de Estadística, Geografía e Informática; 2001). Point cluster analysis of quadrants relied on QUADRAT (IDRISI 32, Clark Labs, Worcester, MA), which determines the total number, mean number or density, variance and variance/mean ratio of points in cases where each grid cell measures the total point count found in that cell. The variance/mean ratio describes the pattern of a point set, with values close to 1.0 suggesting a random point pattern, values significantly smaller than 1.0 suggesting a regularly distributed pattern, and values greater than 1.0 suggesting a clustered pattern. The location of each case was compared with the locations of the reported TB cases. Final display relied on ArcView GIS software (ArcView GIS 3.2, Environmental Systems Research Institute Inc., Redlands, CA).

This study was approved by the Committee of Research Ethics at the Centro de Investigación de Enfermedades Tropicales of the Universidad Autónoma de Guerrero. 

## 3. Results

### 3.1. Spoligotyping Patterns of *M. tuberculosis* Isolates

Spoligotyping the 267 isolates produced 85 distinct genotypes, 59 of them with a unique pattern. The 208 (77.9%) remaining isolates were grouped into 26 clusters that were shared with at least one patient. The cluster size varied from 2 to 70 patients, however, most (21/26) included 2 to 5 patients. The Recent Transmission Index, or RTI*n*, taking into account the number of cases with unique genotypes (singletons), was 0.78, and the RTI*n* − 1 was 0.68.  Table 1 presents a comparison of the Acapulco spoligotypes with data from the global spoligotype database of the cluster genotypes; four were new and 22 had been previously identified by this database. 

Approximately, 59% of the 85 Acapulco spoligotypes, involving 58 (21.7%) of the 267 *M. tuberculosis* isolates, were previously unreported. The main SITs (Spoligo International Type Number)—19, 8, 53, 42, and 342—permitted the classification of isolates in representative patterns described as families or groups [3, 13, 14]. The largest single cluster of 70 patients included 26.2% (70/267) of *M. tuberculosis* isolates of Acapulco; the spoligotype pattern was the East African Indian group (EAI2), previously reported as *M. tuberculosis* Manila family [15]. Approximately 44.6% (119/267) of the isolates descended from East African Indian family (EAI) subgroups 1, 2, 3, and 5. Another 11.6% (31/267) came from Latin American and Mediterranean families (LAM) subgroups 1, 2, 3, 4, 6, and 9. A further 11.2% (30/267) belonged to the “T” family (strain of modern TB) subgroups 1 and 2. Another 3.7% (10/267) were of the “Manu” family, subgroups 1 and 2, and 3.0% (8/267) consisted of the Haarlem family, subgroups 1, 2, and 3. A further 1.9% (5/267) were group “S”, and 1.1% (3/267) were “X” family subgroup 3 and 3 variant 1. Two cases (0.8%) were not classified (“U”), and 0.4% (1/267) pertained to the Bovis group, subgroup 1. We identified no isolate of the Beijing genotype.

### 3.2. Detection of an Emerging Strain and Visualization of the Relations between Spoligotypes

The S19 strain demonstrates an elevated rate of transmission, independent of the sampling fraction *f* (Table 2), behaving as an emerging strain according to the method proposed by Tanaka and Francis [16]. According to the SpolDB4 database, S19 corresponds to the EAI2-Manila strain.

**Figure 1:**
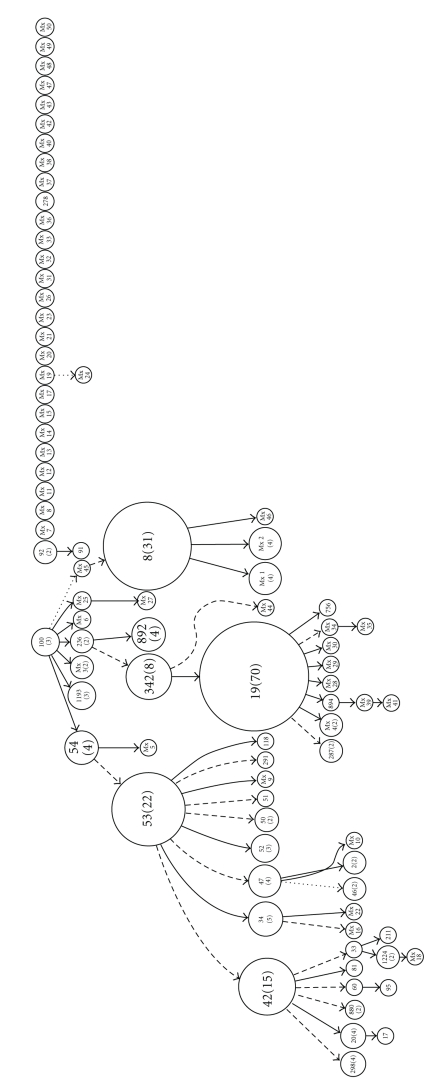
Spoligoforest of tuberculosis cases in Acapulco, Mexico. Nodes are labeled with the ST identifier as indicated in SoplDB4 [3], with the cluster size enclosed in parentheses. Spoligotype not in SpolDB4 was labeled as “Mx” with a number. The size of each node increases as a function of the number of isolates (size of the cluster); the lines among the nodes reflect the evolutionary relationships among spoligotypes with arrows that denote the descendent. If the weight of the line was equal to 1, we drew a solid line; if the weight was greater than or equal to 0.5 but less than 1, we used a dashed line; if the weight was less than 0.5, we used a dotted line.

Figure 1 shows the Spoligoforest hierarchical layout, with the lines between the nodes denoting the weights calculated using this model. The spoligoforest contains three trees with connected components and 26 unconnected nodes. The biggest tree has the ST 100 strain as a root, suggesting that it is the oldest spoligotype in this tree. Seven spoligotypes descend from ST 100, four of which are in small clusters (ST 54, ST 1193, ST 236, and Mx 3, with 4, 3, 2, and 2 isolates, resp.). The ST 54 and ST 236 spoligotypes form two lineages distinct from ST 100. Comparing these spoligotypes with the families in the SpolDB4 database, these lineages are classified as Manu Family and EAI (East African Indian). The Mx 45 genotype, not registered in the SpolDB4 database, also descending from ST 100, originates another lineage. ST 53 and its descendents ST 42, ST 34, ST 47, ST 52, ST 50, ST 51, ST 291, and ST 118 belong to the LAM (Latin American Mediterranean), Haarlem, cluster T and Clade S family of strains. ST 342 and its descendents ST 19 and Mx 44, ST 19 belongs to the Manila family (EAI2-Manila) and is the biggest node, representing 70 isolates and Mx 44 are not registered in SpolDB4. Finally, ST 8 is from the EAI (East African Indian) family, and its descendents Mx 1, Mx 2, and Mx 46 are not registered in SpolDB4.

### 3.3. Epidemiological and Clinical Characteristics of the Tuberculosis Cases

Of 330 diagnosed TB cases in the series, 30 (9.1%) could not be found; 273 (91.0%) of the 300 contacted yielded a positive culture of *M. tuberculosis:* 190 (63.3%) of them were male, and the average age was 41.0 years (standard deviation: 15.5, range: 15–86 years). Most (90.7%, 272/300) were from Guerrero state, and 55.1% (150/272) of these were from the municipality of Acapulco (Table 3). Some 59% (172/293) lived less than 16 years in their community of origin. The average duration of residence in Acapulco was 2.6 years (SD: 1.66, range: 1–10 years). Household had an average of 5.1 occupants (SD: 2.83, range: 1–20 people). 

Data on the start date of symptoms permitted identification of the “first case” (index case) in the largest clusters. The “first case” of the Manila cluster, for example, was a 35-year-old adult addicted to drugs and alcohol who had symptoms for 10 years and, on examination, had advanced pulmonary TB (bacilloscopy positive, grade 3). Thirty of the 70 cases in this cluster reported a concomitant disease, diabetes being the most common (17/70).

**Figure 2:**
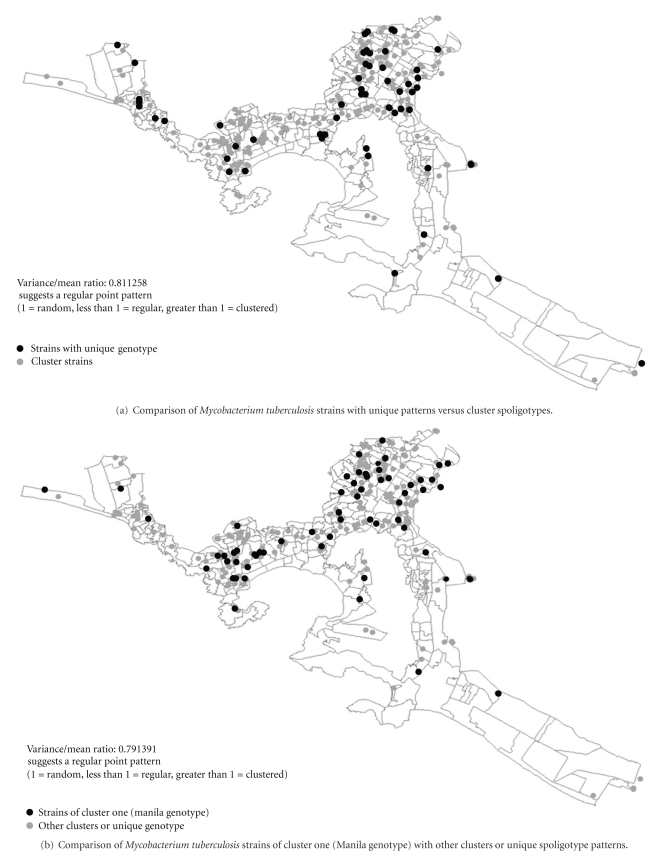
Geographical distribution of tuberculosis cases in Acapulco, Mexico. Mapping and georeferencing of tuberculosis cases used a scale of 1 : 50000. Point cluster analysis relied on the IDRISI QUADRAT module. The variance/mean ratio significantly smaller than 1.0 suggests a regular distribution, while values greater than 1.0 suggest spatial clustering.

The largest five clusters included 70, 31, 22, 15, and 8 cases, together contributing 70% (146/208) of the case series (Table 4).  Figure 2 shows a random distribution pattern of spatial variances. There was no evidence of spatial grouping, consistent with recent infection associated with factors other than residence. 

Table 5 shows the list of conventional TB risk factors, none of which on their own showed a statistically significant association with clustering on univariate analysis. Seven risk profiles, combining two risk factors, did show significant differences between clustered and nonclustered TB cases (Table 6): males who consumed alcohol, unmarried men under the age of 30 years, unemployed single cases, indigenous cases without remunerated employment, young people in urban areas, and uneducated people living in one-roomed dwellings. The size of the study did not permit combining all of these profiles in a single multivariate model. Each was therefore tested to see if it was explained by each other profile in turn. Only one risk profile remained that could not be explained by any others—uneducated cases living in single-room dwellings.

## 4. Discussion

Reflecting the still very partial documentation of spoligotypes worldwide, 58% of our spoligotypes were unique to Acapulco; 21.7% of cases were of a type not previously registered in the global database. 

Our five most frequent spoligotypes included more than one half (54.7%) of the cases; these types are commonly recognized worldwide [3, 13, 14]. The T1 Spoligotype (shared type, 53), EAI (shared type 19, 8, and 342), and LAM9 (shared type 42) currently represent 28.75% of *M. tuberculosis* isolates in the global spoligotype database, being prevalent in Europe, Africa, India, and other countries [3].

The largest cluster, including 33.6% (70/208) of the isolates, was the Manila family. This demonstrates the public health impact of this strain in Acapulco. This relates to Mexico's historic ties with Asia, as the Manila strain is found throughout South East Asia, particularly in the Philippines (73%), Myanmar and Malaysia (53%), and Vietnam and Thailand (32%) [3, 15, 17]. In a study in the Philippines, 90% (43/48) of isolates were of the Manila genotype [15]. Phylogenetic studies of related spoligotype strains demonstrated that the EAI (East African Indian) genotype has shared ancestral relations [17]. This group includes the Manila or EAI2-Manila strain [3]. 

Internationally, certain *M. tuberculosis* strains are linked with a large proportion of recent infection, suggesting these strains might have greater transmissibility or higher probability to cause disease soon after transmission. These strains are associated with families or groups of related isolates, such as the genotypes of the Beijing family strain W, Haarlem, and Africa [3, 18, 19]. These “cluster strains” come from the Latin American Mediterranean families (LAM), Haarlem (H), and *M. Bovis* [20]. The Manila strain, responsible for a third of our cluster strain cases, might share this greater transmission or progress to active disease—at least in Acapulco. 

The method used for detection of rapidly propagating strains [16] allowed identification of an emerging strain (strain S19), previously classified as EAI2-Manila [3]. This result coincides with the spoligoforest result, where node S19 (*n* = 70) has the most rapid transmission. Spoligoforest demonstrates all of the possible relations between spoligotypes under the assumption of spoligotype mutation [12]. In our data set, the largest root of the tree was the ST 100 strain, which corresponds to the MANU family. Among the principal descendants, we identified strains ST 54, ST 236, and ST 1193. However, the majority of the 26 unconnected nodes had not been previously registered in the SpolDB4 database. 

Our proportion of clustered strains (77.9%) is higher than in other studies. A six-year study in two urban communities in South Africa identified 72% of the cases as belonging to clusters [21]. Similar results came from the Grand Canary Islands of Spain [4]. Other studies in South Africa and in Equatorial Guinea identified a level of grouping of 67 and 61.6%, respectively [5, 22]. A range of clustering rates (31 to 67.7%) have been reported in cities from industrial countries such as Spain, Italy, Holland, Denmark, Slovenia, Canada, and United States [23–29], while France, London, and Switzerland reported a minor portion of 18 to 27.6% cluster strains [30–32]. 

Considering the probable existence of an index case in each cluster, we estimated that 68% (208–26) of our 267 cases could have been due to recent infection [10]. Taking into account the number of singletons, the estimate of RTI was greater (78%). Migration, duration of the study [33], predominance of a local strain, simultaneous reactivation of an infection acquired remotely, and laboratory error can all influence the reliability of this inference [34]. In our study, other factors support a high proportion of recent transmission, including the high prevalence rate, the mobility of population [29], the bacillary load of the cases, and the duration of their symptoms.

Although one cluster included 70 cases, most clusters had five or fewer cases. Similar results come from the Grand Canary Islands [4]. A study in San Francisco in 1996-97 found 73 of 221 (33%) cases in multiple clusters, implying multiple points of infection in the community. 

Analysis of why certain strains of *M. tuberculosis* propagated effectively points mostly to delayed diagnosis [35]. The index cases in five of our clusters were young adult males with at least one risk factor, such as drug use or alcohol addiction [4, 10, 25, 36]. Cases with an associated pathology have a higher tendency to acquire a new infection of *M. tuberculosis* [37] or to develop tuberculosis [38].

Our analysis of potential risk factors for clustering did not produce clearly actionable results. The notable absence of spatial clustering implies that place of residence is not a useful risk indicator. Type of residence (single room) combined with lack of formal education, on the other hand, was the single enduring risk profile. 

SpolTools permitted the analysis of *M. tuberculosis *spoligotyping data to identify emerging strains and visualization of the probable evolutionary relationships between the spoligotypes in our series. Considering that a single emerging strain, the EAI2-Manila genotype, can account for so many cases, an evolution of spoligotyping could conceivably be used to evaluate the impact of TB prevention and early diagnosis efforts. Further studies of the virulence and drug sensitivity of the Manila genotype may be warranted.

##  Conflicts of Interests

The authors do not have funding, commercial or other associations that might pose a conflict of interest.

## Figures and Tables

**Table 1:** Acapulco patterns of *Mycobacterium tuberculosis* spoligotypes.

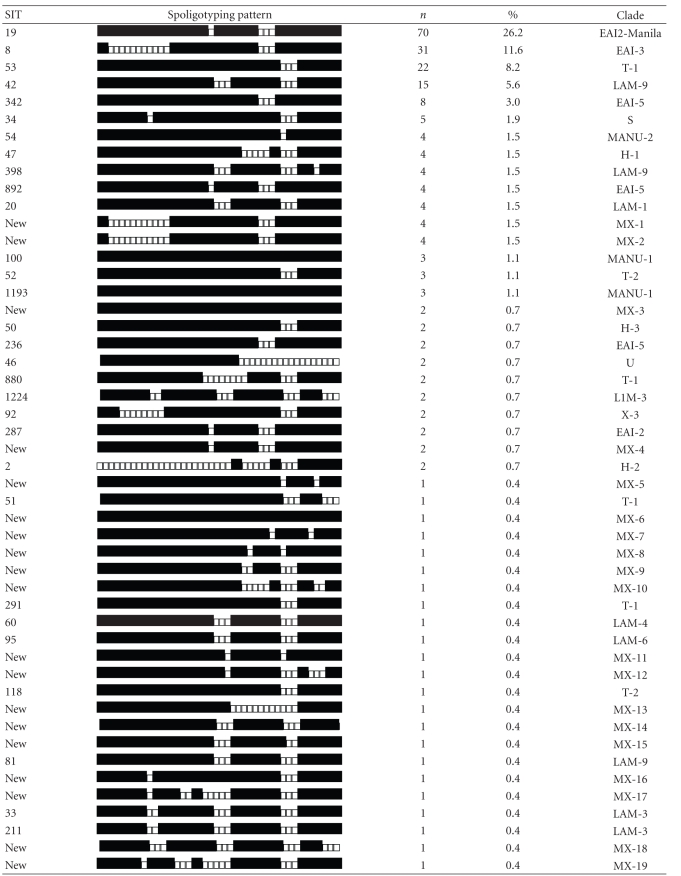 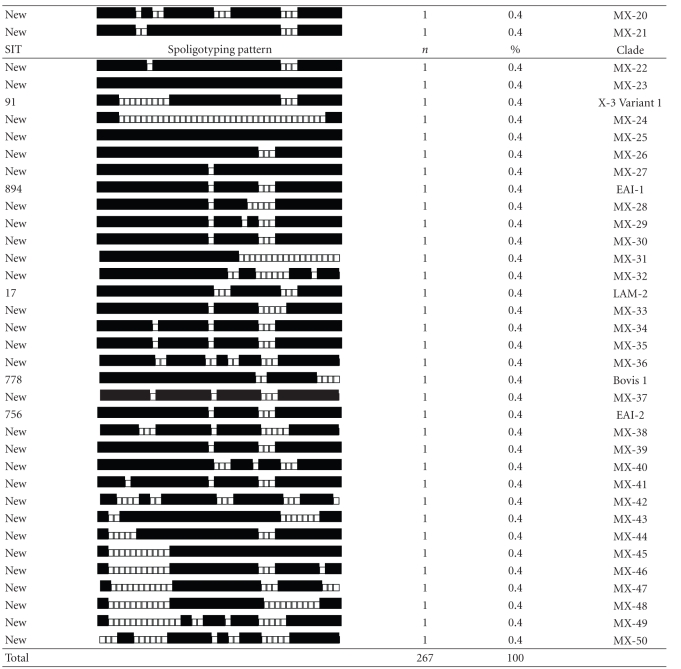

SIT, designation of the spoligotype in the international database. Spoligotyping patterns, binary description: (■) hybridization and (□) no hybridization. *n*, number of strains; clade, defined mainly as described [4, 5]: EAI-2/Manila: East African Indian 2/Manila, T (ill-defined T cluster), LAM: Latin American and Mediterranean, S: S clade, MANU: Manu family, H: Haarlem, U: Undesignated, X: X cluster, Bovis. New clusters designated as MX (Mexican).

**Table 2:** Emerging strains detected in the pulmonary TB by using spoligotypes in Acapulco.

*f*							
0.95		0.5		0.1		0.01	
Strain	q value	Strain	q value	Strain	q value	Strain	q value

S19	0.00000000	S19	0.00000006	S19	0.00000477	S19	0.00001087
S8	0.03205437	S8	0.08223924	S8	0.17184278	S8	0.20014382

*f = *sampling fraction.

**Table 3:** Characteristics of the pulmonary TB case series in Acapulco.

Variable	*n* = 300	%
Sex*		
Male	190	63.3
Female	110	36.7
Age (in years)**		
15–24	51	17
26–34	72	24
35–44	58	19.3
45–54	55	18.3
55–64	40	13.3
65 +	24	8
*Signs and symptoms *		
Duration of the cough >3 months**	192	64
Lost weight**	271	90.3
Fever**	227	75.7
Hemoptysis**	151	50.3
Associated illness**	132	44
Type of associated illness** (n = 132)		
Diabetes	77	58.3
Drug user	20	15.2
AIDS	12	9.1
Alcoholism	7	5.3
Undernutrition	3	2.3
Others	13	9.8
Prior TB**	26	8.7
Husband/wife with prior TB** (n = 190)	22	11.6
Recent contact with someone with TB**		
Yes	112	37.3
No	150	50
Do not know	37	12.3
Indigenous**	27	9
Indigenous group** (n = 27)		
Náhuatl	16	59.3
Mixteco	9	33.3
Amuzgo	2	7.4
State of birth**		
Guerrero	272	90.7
Oaxaca	11	3.7
Estado de México	4	1.3
Michoacán	3	1
Other state	10	3.3
Region of birth** (n = 272)		
Acapulco	150	55.1
Zone Centro	25	9.2
La Montaña	7	2.6
Costa Chica	46	16.9
Costa Grande	21	7.7
Zone Norte	8	2.9
Tierra Caliente	12	4.4

*P value =.05,

**P value <.05.

**Table 4:** Characteristics of the largest clusters detected by spoligotyping in Acapulco.

No. Cluster	Cluster size (Number of male)	Age (range)	Place of birth (*n*)	Indigenous group (*n*)	Associated illness % (*n*)	Immune- compromised illness (*n*)	Previous TB % (*n*)	Contact/ TB case % (*n*)	Husband/ wife with TB % (*n*)	BAAR diagnostic %
1	70 (45)	41.6 (15–86)	Guerrero (62) Oaxaca (4) Mexico (1) DF (1) Gto (1) Tabasco (1)	Nah (4)	43% (30)	Diabetes (17) AIDS (2) Alcoholism (2) Drug user (2) undernourished (2)	10% (7)	39% (27)	8% (5)	+ 47 ++ 23 +++ 30

2	31 (21)	41.2 (21–73)	Guerrero (28) Oaxaca (1) EdoMex (1) Veracruz (1)	Nah (2)	45% (14)	Diabetes (10) AIDS (1)	13% (4)	26% (8)	17% (5)	+ 36 ++ 19 +++ 45

3	22 (19)	44.0 (22–76)	Guerrero (20) Oaxaca (1) EdoMex (1)	Nah (3) Mix (1)	27% (6)	Diabetes (4) Alcoholism (1) Drug user (1)	14% (3)	41% (9)	0	++ 36 +++ 64

4	15 (7)	45.4 (19–75)	Guerrero (13) Oaxaca (1) Michoacán (1)	Nah (1) Mix (2)	60% (9)	Diabetes (3) AIDS (1) Alcoholism (1) Drug user (3)	0	53% (8)	0	+ 33 ++ 27 +++ 40

5	8 (4)	43.6 (23–68)	Guerrero (8)	Mix (2)	50% (4)	Diabetes (1) Drug user (2)	0	63% (5)	0	+ 12.5 +++ 88

Place of birth: DF= Federal district, Mexico; Gto= Guanajuato state, EdoMex= State of México. Indigenous group: Nah= Náhuatl, Mix= Mixteco.

**Table 5:** Bivariate analysis of conventional TB risk factors, contrasting clustered and nonclustered cases.

Variable		Clustered		Not clustered		OR	95% CI
		*N*	%	*n*	%		
Sex	Male	135	80.6	32	19.4	1.60	0.87–2.2
	Female	72	72.7	27	27.3	1	
Age	<30 years	55	76.4	17	23.6	0.91	0.48–1.72
	≥30 years	150	78.1	42	21.9	1	
Marital status	Single	65	83.3	13	16.4	1.64	0.83–3.2
	Married	140	75.3	46	24.7	1	
Education	No formal studies	39	72.2	15	27.8	0.67	0.34–1.33
	Formal study	166	79.4	43	20.6	1	
Occupation	Unemployed	70	74.5	24	25.5	0.76	0.40–1.43
	Employed	135	79.4	35	20.6	1	
Ethnicity	Indigenous	19	73.1	7	26.9	0.75	0.28–2.08
	Mestizo	189	78.4	52	21.6	1	
Migration	Yes	99	76.2	31	23.8	0.82	0.45–1.47
	No	105	79.5	27	20.5	1	
Area of residence	Urban	153	78.5	42	21.5	1.19	0.59–2.37
	Rural	52	75.4	17	24.6	1	
Duration of residence	≥17 years	123	79.4	32	20.6	1.09	0.59–2.0
	<17 years	74	77.9	21	22.1	1	
Number of residents per household	≥6 persons	70	72.9	26	27.1	0.66	0.37–1.19
	<6 persons	135	80.4	33	16.6	1	
Use of alcohol	Yes	67	75.3	22	24.7	0.82	0.45–1.50
	No	137	78.7	37	21.3	1	
Concomitant illness	Yes	94	77.7	27	23.3	1.02	0.55–1.91
	No	111	77.6	32	23.4	1	
Spouse with TB	Yes	15	71.4	6	28.6	0.66	0.24–1.85
	No	113	79.0	30	21.0	1	

**Table 6:** Bivariate analysis of risk profiles associated with clustering.

Variable		Cluster		No cluster		OR	95% CI
		*N*	%	*n*	%		
Among cases who used alcohol	Male	71	85.5	12	14.5	2.1	1.05–4.03
	Female	68	73.1	25	26.9	1	
Among cases over the age of 29 years	Single	36	92.3	3	7.7	4.1	1.29–13.1
	Married or cohabiting	114	74.5	39	23.5	1	
Among cases single/unmarried cases	Unemployed	7	87.5	1	12.5	13.7	1.81–104
	Employed	8	40.0	12	60.0	1	
Among cases with remunerated employment	Mestizo	132	81.0	31	19.0	3.2	1.07–9.1
	Indigenous	8	57.1	6	42.9	1	
Among cases who live in one place for less than 15 years	Urban	66	78.6	18	21.4	3.0	1.22–10.0
	Rural	11	55.0	9	45.0	1	
Cases younger than 30 years of age	Urban	43	82.7	9	17.3	3.2	1.03–9.82
	Rural	12	60.0	8	40.0	1	
Among cases without formal education	1 room	22	91.7	2	8.3	8.9	2.0–39.1
	2 or more rooms	16	55.2	13	44.8	1	

OR= Odds Ratio, CI= Confidence Interval.
